# *Saccharomyces jurei* sp. nov., isolation and genetic identification of a novel yeast species from *Quercus robur*

**DOI:** 10.1099/ijsem.0.002013

**Published:** 2017-06-22

**Authors:** Samina Naseeb, Stephen A. James, Haya Alsammar, Christopher J. Michaels, Beatrice Gini, Carmen Nueno-Palop, Christopher J. Bond, Henry McGhie, Ian N. Roberts, Daniela Delneri

**Affiliations:** ^1^​ Manchester Institute of Biotechnology, Faculty of Biology, Medicine and Health, The University of Manchester, Manchester M1 7DN, UK; ^2^​ Institute of Food Research, Norwich, Norfolk, UK; ^3^​ The Manchester Museum, The University of Manchester, Manchester M13 9PL, UK

**Keywords:** *Saccharomyces*, yeast, new species, *Saccharomyces jurei*, isolation protocol

## Abstract

Two strains, D5088^T^ and D5095, representing a novel yeast species belonging to the genus *Saccharomyces* were isolated from oak tree bark and surrounding soil located at an altitude of 1000 m above sea level in Saint Auban, France. Sequence analyses of the internal transcribed spacer (ITS) region and 26S rRNA D1/D2 domains indicated that the two strains were most closely related to *Saccharomyces mikatae* and *Saccharomyces paradoxus*. Genetic hybridization analyses showed that both strains are reproductively isolated from all other *Saccharomyces* species and, therefore, represent a distinct biological species. The species name *Saccharomyces jurei* sp. nov. is proposed to accommodate these two strains, with D5088^T^ (=CBS 14759^T^=NCYC 3947^T^) designated as the type strain.

## Introduction

The *Saccharomyces sensu stricto* group is composed of eight biologically distinct yeast species, namely *Saccharomyces cerevisiae*, *S. paradoxus*, *S. cariocanus*, *S. uvarum*, *S. mikatae*, *S. kudriavzevii, S. arboricola* and *S. eubayanus* [1–6], and two natural hybrids, namely *S. pastorianus* [7, 8] and *S. bayanus* [9]. *S. cariocanus* was initially included in the genus based on karyotyping and reproductive isolation [3]. However, subsequent genome sequence analysis of the only two known strains (of *S. cariocanus*) showed them to belong to one of three geographically well-defined populations of *S. paradoxus* (i.e. American population) [10, 11]. The most recent phylogenetic analyses of the genus excluded both *S. cariocanus* and *S. bayanus*, the latter due to it being of hybrid origin [12, 13]. The cryotolerant yeast *S. eubayanus* is the latest addition to the genus. This species was first isolated in *Nothofagus* (southern beech) forests in Patagonia, Argentina [6], but has since been found in North America, on the Tibetan Plateau and most recently on the North Island of New Zealand [14–16].

The *Saccharomyces* species are defined by the biological species concept since they are reproductively isolated via postzygotic barriers [3, 10, 17]. All of these species possess typical budding shape morphology, have the same number of 16 chromosomes [18], and they can be differentiated from one another based on the sequences of their internal transcribed spacer (ITS) and 26S rRNA D1/D2 regions [19, 20]. It has been shown that the majority of yeast species can be identified from sequence divergence of the D1/D2 domain [21]. Sequencing of the ITS1 and D1/D2 regions is therefore routinely used for identifying yeast strains [3, 22–25].

*Saccharomyces* yeasts have been isolated from a wide variety of different substrates including deciduous tree bark, surrounding soil, tree exudates (sap), fruits, insects and vineyard grapes [2, 26–28]. *S. paradoxus* is the most commonly isolated species in nature and has been found globally from natural resources, and most notably from oak trees (*Quercus* spp.) and surrounding soil [11, 29]. Moreover, *S. cerevisae* and *S. paradoxus* have been isolated from the same locations, indicating that populations of the two species coexist in nature [30, 31]. *Saccharomyces* hybrids have been often isolated from domesticated environments such as vineyards [32] and breweries, and are known to be associated with fermentation processes for the production of wine and beer. The best example of this is the lager yeast *S. pastorianus* (syn. *S. carlsbergensis*), a cold-adapted *S. cerevisiae* × *S. eubayanus* alloploid hybrid [6].

To date, most of the *Saccharomyces* strains held in international yeast collections (e.g. the Westerdijk Fungal Biodiversity Institute, CBS) have been isolated from substrates collected and sampled at low altitudes [26, 30, 33, 34]. Some species found from higher altitude include *S. eubayanus* isolated from the Tibetan Plateau [14] and *S. arboricola* from the Qinling Mountains [2]. Consequently, very little is known about the ecology and geographical distribution of *Saccharomyces* yeasts found at higher altitudes and cooler conditions. Thus, sampling substrates such as soil and trees at higher altitudes may lead to the discovery of new cryotolerant yeast strains and species. In this study, we sampled oak tree bark and surrounding soil at an altitude of 1000 m above sea level in Saint Auban, France. The yeast community was isolated and the species identities were determined by standard ITS and D1/D2 sequencing. Whilst the majority of isolated *Saccharomyces* were identified as *S. paradoxus*, two strains (D5088^T^ and D5095) were recovered and found to represent a novel species belonging to the genus *Saccharomyces*. The novel species is named *Saccharomyces jurei* sp. nov., in memory of the yeast researcher Professor Jure Piškur. We show here that *S. jurei* is reproductively isolated from other *Saccharomyces* species by performing genetic crosses and testing for hybrid sterility. Both strains formed viable hybrids with all other *Saccharomyces* species and were, as expected from crosses between different biological species, predominantly sterile (with a spore viability ranging from 0 to 3 %).

## Methods

### Yeast isolation, media and maintenance

Samples of bark and soil were obtained in July 2013 from oak trees (*Quercus*) growing at an altitude of 1000 m above sea level in the Saint Auban region of south-eastern France (43° 5.2′ N 006° 44′ E). The samples were collected aseptically and stored in sterile bags or Petri dishes. Equal amounts of each bark and soil sample were independently placed into one of two 50 ml sterile Falcon tubes containing Sniegowski enrichment medium consisting of 3 g yeast extract, 3 g malt extract, 5 g peptone, 10 g sucrose, 76 ml EtOH, 1 mg chloramphenicol and 1 ml of 1 M HCl per litre [31]. The Falcon tubes were tightly capped and one set was incubated (without agitation) at 30 °C, while the other was incubated at 20 °C for 20–25 days. The tubes were periodically examined for turbidity and fermentation (i.e. evidence of gas formation). These observations were done after 10 days for the samples incubated at 30 °C and after 20 days for the 20 °C samples. Samples showing signs of either turbidity or fermentation were further examined, for signs of yeast growth, by standard light microscopy. All samples positive for yeast growth were plated onto Sniegowski selection medium (SSE) [31] and incubated at 30 °C for several days. Individual yeast colonies were picked and re-streaked onto fresh SSE plates for further characterization.

### Morphological and physiological characterization of yeasts

The two strains were characterized biochemically, morphologically and physiologically according to standard methods described previously [35]. Growth temperature was determined by cultivation on YM (yeast extract-malt extract) agar. Sporulation tests were performed on cornmeal agar, Gorodkowa agar, potassium acetate agar and YM agar, and plates were incubated at 25 °C for 3–4 weeks in individual and mixed cultures.

Images of the asci were taken using an Olympus model BH-2 light microscope and a scanning electron microscope. The asci formed on acetate agar after 5 days at 25 °C, and spontaneously broke as result of the general fixing process.

### DNA extraction

For ITS1 and 26S rRNA D1/D2 sequencing, genomic DNA was isolated from cultures freshly grown on plates using the Masterpure Yeast DNA extraction kit (catalogue no. MPY80200) and following the manufacturer’s protocol. DNA yields and *A*_260_/*A*_280_ ratios were measured using a Nanodrop spectrophotometer (ND-1000), while DNA purity and integrity were checked by 0.8 % agarose gel electrophoresis.

### DNA sequencing

The variable D1 and D2 domains of the 26S rRNA gene were amplified and sequenced using primers NL1 and NL4 [36]. The ribosomal ITS region was amplified using primers ITS4 and ITS5, and sequenced using these primers as well as internal primers ITS2 and ITS3 [20, 37]. Translation EF-1αA (*TEF1*) and *RPB2* genes were amplified and sequenced as described previously [23]. Other nuclear genes (*CAT8*, *CYR1*, *GSY1*, *MET6* and *OPY1*) were amplified and sequenced using previously published primers [32]. The PCR fragments were analysed by standard 1 % agarose gel electrophoresis, purified and concentrated using QIAquick PCR purification spin columns (Qiagen) following the manufacturer’s instructions. The purified products were sequenced using the BigDye Terminator Ready Reaction kit, version 3.1 (Applied Biosystems) following the manufacturer’s instructions. Sequence traces were edited manually, and consensus sequences were generated using the program seqman, version 11 (DNASTAR). The sequences were compared pairwise using a fasta similarity search [38] and were aligned with the sequences of closely related taxa, retrieved from the EMBL sequence database, using the multiple alignment program clustal w [39] included in the mega version 6 software package [40]. A phylogenetic tree was reconstructed from the combined sequences of the 26S rRNA D1/D2 and ITS regions (including the 5.8S rRNA) using the neighbour-joining (NJ) program [41] included in mega, with the Kimura two-parameter (K2P) distance measure and *Naumovozyma castellii* selected as the outgroup species. Bootstrap support for the NJ tree was determined from 1000 replicates.

### Construction of stable haploid strains possessing auxotrophic marker

Prototrophic diploid strains D5088^T^ and D5095 of *S. jurei* were made heterothallic by knocking out the *HO* gene. The heterozygote *HO*/*hoΔ* diploid strains were sporulated and tetrads were dissected to obtain stable *Mat***a** and *Matα hoΔ* haploid strains. The mating types were determined by PCR as described previously [42]. A PCR-mediated gene deletion strategy using drug resistance cassettes was applied to generate *ura3* auxotrophic strains of *S. jurei* [43]. A standard PEG/LiAc heat-shock protocol with some modifications was used for transformation. In our modified protocol, 1.0–3.0 µg of PCR product was transformed and cells were incubated at 30 °C for 30 min followed by heat-shock at 37 °C for 20 min. For the selection of transformants, the cells were incubated overnight at room temperature before being plated on selective media. The verification of gene deletions was performed by diagnostic colony PCR using gene-specific and cassette-specific primers.

### Spore viability analysis

*S. jurei* strains D5088^T^ and D5095 were crossed with other species of *Saccharomyces* using a micromanipulator. The hybrids were selected on SD plates containing different selective markers [44]. The tetrads were formed by growing the hybrids in pre-sporulation medium at 30 °C for 12 h before plating on minimal sporulation medium. The sporulation plates were incubated at 20 °C for 7–10 days for the formation of tetrads. The tetrads were dissected using a Singer MSM-300 micromanipulator. Spore viability was calculated based on the percentage of viable spores that had grown for each variant of the strain out of a possible 64 dissected tetrads.

## Results and Discussion

### Isolation of yeast species from *Quercus robur*

We obtained a total of 284 yeast isolates from oak tree bark and soil samples incubated at 20 and 30 °C (see Table S1, available with the online Supplementary Material). *S. paradoxus* was by far the most abundant species isolated from the bark and soil samples, with *Kazachstania servazzii* and *Lachancea* (*Kluyveromyces*) *thermotolerans* being the other species recovered from this site.

### DNA sequencing and phylogenetic analysis

**Fig. 1.**
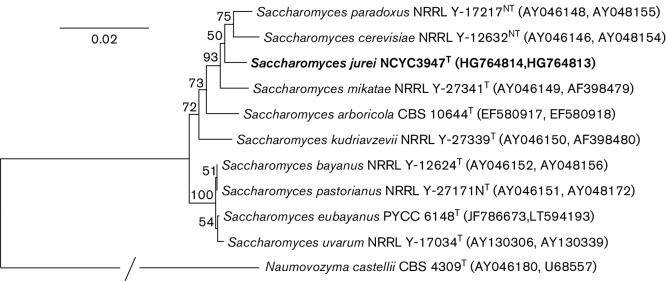
NJ dendrogram based on the combined sequences of the LSU D1/D2 and ITS regions (including 5.8S rRNA) of *Saccharomyces jurei* sp. nov. and its closest relatives. Species names are followed by CBS, NCYC, NRRL or PYCC strain accession numbers and, respectively, the EMBL/GenBank accession numbers for the ITS and LSU D1/D2 regions. *Naumovozyma castellii* was used as the outgroup species for the analysis. Boostrap values of >50 %, determined from 1000 replicates, are shown at branch nodes. Bar, 2 base substitutions per 100 nt.

All yeast isolates were initially screened by amplifying the ITS region to distinguish between *Saccharomyces* and non-*Saccharomyces* species based on differing fragment size. Of 284 isolates collected, 180 amplified ITS fragments of the correct size for *Saccharomyces* yeasts (~850 bp), and their species identities were confirmed by sequencing the ITS1 region. Although the majority of isolates were identified as representing *S. paradoxus* (172 isolates), two isolates, D5088^T^ and D5095, had ITS1 sequences that did not match with any currently described *Saccharomyces* species. Both isolates had identical ITS1 sequences, and a fasta sequence similarity search of the EMBL fungal sequence database revealed no other yeast taxon, either *Saccharomyces* or non-*Saccharomyces*, with an ITS1 sequence identical to these isolates. In terms of pairwise sequence similarity, the closest taxa were *S. mikatae* (98.1 %; 7 nt substitutions in 360 nt) and *S. paradoxus* (96.1 %; 12 nt sustitutions and one indel in 362 nt). Indeed, an ITS1 sequence alignment of the novel *Saccharomyces* taxon, *S. cerevisiae*, *S. mikatae* and representatives of the three geographically distinct populations of *S. paradoxus* (i.e. North American, European and Far Eastern) confirmed that the ITS1 region of strains D5088^T^ and D5095 was unique and possessed four species-specific single nucleotide polymorphisms (Fig. S1). The level of sequence similarity seen between *S. jurei* and *S. paradoxus* is comparable to that observed between *S. mikatae* and *S. paradoxus* (96.1 % versus 96.4 %). Furthermore, the level of sequence similarity between *S. jurei* and *S. mikatae* (98.1 %) is lower than that observed between the three *S. paradoxus* populations (99.2–99.7 %). In contrast to the ITS1 region, a fasta sequence similarity search with the 26S rRNA D1/D2 sequence revealed that the closest known taxon was *S. paradoxus* (99.8 %; 1 nt substitution in 579 nt), with *S. mikatae* displaying only 98.6 % similarity (6 nt substitutions and one indel in 574 nt). Sequence analysis of seven other nuclear genes (*CAT8*, *CYR1*, *OPY1*, *GSY1*, *MET6*, *TEF1* and *RPB2*) showed that *S. jurei* is divergent from *S. cerevisiae*, *S. mikatae* and *S. paradoxus* (Table S2). Moreover, different populations of *S. paradoxus* and *S. cerevisiae* possess approximately 98–99 % sequence similarity for the seven nuclear genes analysed in this study. A phylogenetic analysis based on the combined (i.e. concatenated) sequences of the ITS and 26S rRNA D1/D2 regions showed that the novel taxon [as represented by D5088^T^ (=NCYC 3947^T^)] belonged to the genus *Saccharomyces*, and is located between *S. mikatae* and the species pair of *S. cerevisiae* and *S. paradoxus* (Fig. 1).

### Genetic hybridization analysis

All eight members of the *Saccharomyces* genus are biological species since they are reproductively isolated from each other [3]. The species of this genus can readily hybridize with each other, although the interspecific hybrids (F1 hybrids) formed are sexually sterile [45, 46]. This sterility is caused by the inability of the two diverged homologous chromosomes to recombine during meiosis [17, 47, 48]. The presence of chromosomal rearrangements also lowers spore viability and contributes to hybrid infertility [10, 49, 50]. In contrast, intraspecifc *Saccharomyces* hybrids are fertile and yield highly viable ascospores [3]. To establish that *S. jurei* is a novel biological species of the genus, we performed direct genetic crosses with representative strains from all other *Saccharomyces* species and tested the fertility of the resulting hybrids.

We first analysed the fertility of strains D5088^T^ and D5095. Both were observed to be homothallic and highly fertile, with ascospore viability ranging from 95 to 100 %. To test the fertility of intra- and interspecific hybrids, we successfully constructed genetically stable haploid strains (**a** and α mating types) of D5088^T^ and D5095 possessing *ura3* auxotrophy and drug resistance markers (Clonat and KanMX, respectively). The hybrids produced from crossing D5088^T^ with D5095 showed high spore viability of ca. 89 %, confirming that both strains belong to the same biological species (Table 1). In contrast, the interspecific hybrids produced from crosses between *S. jurei* and the other *Saccharomyces* species although viable displayed extremely low spore viability, ranging from 0 to 0.3 % (Table 1). Collectively, these data confirm the post-zygotic isolation between *S. jurei* and the other member species of the genus *Saccharomyces*, and demonstrate that it represents a biologically distinct novel *Saccharomyces* species.

**Table 1.** Genetic identification of hybrids between *S. jurei* (D5088^T^ and D5095), *S. cerevisiae* (FY3), *S. mikatae* (IFO 1815^T^), *S. paradoxus* (N-44, N-17, YPS138), *S. uvarum* (CBS 7001), *S. kudriavzevii* (IFO 1802^T^), *S. arboricola* (CBS 10644^T^) and *S. eubayanus* (PYCC 6148^T^)

**Hybrids engineered**	**No. of tetrads dissected**	**Percentage of viable ascospores of hybrids**
*S. jurei*×*S. jurei*		
D5095×D5088^T^	640	89
*S. jurei*×*S. cerevisiae*		
D5095×FY3	624	0.30
D5088^T^×FY3	612	0.50
*S. jurei*×*S. mikatae*		
D5095×IFO 1815^T^	648	2.0
D5088^T^×IFO 1815^T^	472	1.0
*S. jurei*×*S. paradoxus* N-44		
D5095×N-44	484	0
D5088^T^×N-44	648	0.3
*S. jurei×S. paradoxus* N-17		
D5095×N17	532	1.6
D5088^T^×N-17	648	0
*S. jurei×S. paradoxus* YPS138		
D5095×YPS138	640	1.2
D5088^T^×YPS138	600	0.25
*S. jurei×S. uvarum*		
D5095×CBS 7001	604	0.3
D5088^T^×CBS 7001	644	0.2
*S. jurei*×*S. kudriavzevii*		
D5095×IFO 1802^T^	620	0
D5088^T^×IFO 1802^T^	614	1
*S. jurei*×*S. arboricola*		
D5095×CBS 10644^T^	632	0
D5088^T^×CBS 10644^T^	648	0
*S. jurei*×*S. eubayanus*		
D5095×PYCC 6148^T^	632	0.2
D5088^T^×PYCC 6148^T^	648	0

### Phenotypic characterization

**Fig. 2.**
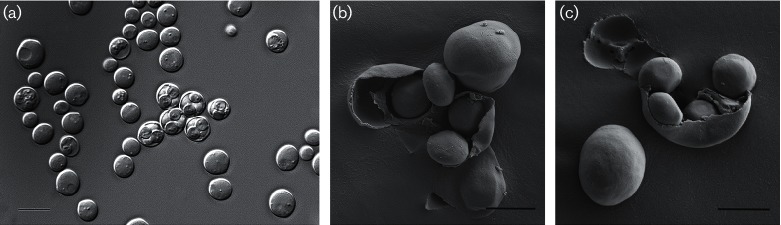
Phenotypic chatacteristics of *Saccharomyces* *jurei* sp. nov. D5088^T^. Photomicrograph of the asci (a) and scanning electron micrographs of ascospores (b and c) formed on acetate agar after 5 days at 25 °C. Bars, 2 µm

Strains D5088^T^ and D5095 exhibited similar morphological and physiological characters that are typical for species belonging to the genus *Saccharomyces* [51]. Moderate sporulation was observed for both strains at 25 °C on potassium acetate agar, cornmeal agar and YM agar. The asci had a spherical shape with two to four oval spores per ascus (Fig. 2).

Phenotypically, as shown in Table S3, there appear to be no standard assimilation or fermentation tests which can be used reliably to differentiate between *S. jurei* and its closest relatives, namely *S. cerevisiae*, *S. mikatae* and *S. paradoxus*. Amongst these four *Saccharomyces* species, *S. cerevisiae* and *S. paradoxus* are the only ones which are able to grow at the elevated temperature of 37 °C, although this trait is somewhat strain-variable [51, 52]. At present, the species descriptions of *S. jurei* (this study) and *S. mikatae* [3, 52] are restricted to just two strains each. However, it is quite possible that in time, as additional strains of each of these species are discovered, some of the traits currently listed as positive (e.g. maltose assimilation and fermentation; Table S3) will be found to be variable, as is the case for both *S. cerevisiae* and *S. paradoxus* [51–55]. The molecular comparisons showed that strains D5088^T^ and D5095 represent a novel species of the genus *Saccharomyces*, for which the name *Saccharomyces jurii* sp. nov. is proposed.

## Description of *Saccharomyces jurei* Naseeb S, James SA, Alsammar H, Michaels C, Gini B, Neuno-Palop C, Bond CJ, McGhie H, Roberts IN, Delneri D., sp. nov.

*Saccharomyces jurei* (ju’re.i. N.L. gen. n. *jurei* in memory of Professor Jure Piškur for his considerable contribution to the fields of yeast genetics and molecular biology).

On YM agar, after 3 days incubation at 25 °C, colonies are light cream-coloured, slightly shiny, smooth and with an entire margin. In YM broth, after 2 days of incubation at 25 °C, cells are spherical to ovoid (5.0–8.0×6.0–10.0 µm) and occur singly or in pairs. Budding is multipolar. No pseudohyphae are observed in cultures grown on cornmeal agar or potato agar. Oval asci containing 2–4 smooth round ascospores are formed after incubation for 1–3 weeks at 25 °C on cornmeal agar, potassium acetate agar and YM agar (Fig. 2). Asci are persistent. Glucose, galactose, sucrose, maltose, raffinose, melizitose and methyl α-d-glucoside are fermented, but not lactose, trehalose, melibiose, cellobiose, inulin, soluble starch or d-xylose. Glucose, sucrose, raffinose, galactose, trehalose (latent or weak), maltose, melezitose, methyl α-d-glucoside, ethanol, glycerol (latent), d-mannitol and dl-lactate are assimilated. No growth occurs on inulin, melibiose, lactose, soluble starch, cellobiose, salicin, l-sorbose, l-rhamnose, d-xylose, l-arabinose, d-arabinose, d-ribose, methanol, erythritol, ribitol, xylitol, galactitol, d-glucitol, inositol, succinate or citrate. No growth occurs on cadaverine, lysine, ethylamine hydrochloride or nitrate. Growth occurs at 30 °C, but not at 37 °C. No growth occurs on either YM agar with 10 % (w/v) NaCl or on 100 µg cycloheximide ml^−1^. Growth occurs on 50 % glucose/yeast extract. Starch-like compounds are not produced.

The type strain, D5088^T^, was isolated from north-facing oak bark, collected at an altitude of 1000 m above sea level in the Saint Auban region of south-eastern France. This strain has been deposited in the National Collection of Yeast Cultures (NCYC), Norwich, UK, as NCYC 3947^T^ (=CBS 14759^T^), and is stored in a metabolically inactive form in accordance with the Code. Strain D5095 has also been deposited in the NCYC as NCYC 3962. The MycoBank deposit number is MB 819910.

## Supplementary Data

166 Supplementary File 1
