# Supplementary material for: Saccharomyces jurei sp. nov., isolation and genetic identification of a novel yeast species from Quercus robur
Source: Int J Syst Evol Microbiol. 2017 Jun 22;67(6):2046–52. doi: 10.1099/ijsem.0.002013 (PMC5817255; doi:10.1099/ijsem.0.002013)
Supplement: Supplementary File 1 [file ijsem-67-2046-s001.pdf]

***Saccharomyces jurei* sp. nov., Isolation and Genetic Identification of a Novel Yeast Species from *Quercus robur*.**

Samina Naseeb[1#], Stephen A. James[2#], Haya Alsammar[1], Christopher Michaels[1], Beatrice Gini[1], Carmen Nueno-Palop[2], Christopher J. Bond[2], Henry McGhie[3], Ian N. Roberts[2] and Daniela Delneri[1*]

International Journal of Systematic and Evolutionary Microbiology

Author affiliations

[1]Manchester Institute of Biotechnology, Faculty of Biology, Medicine and Health, The University of Manchester, Manchester, M1 7DN, UK.

[2]Institute of Food Research Norwich, Norfolk, United Kingdom

[3]The Manchester Museum, The University of Manchester, Manchester, M13 9PL

[#] These authors contributed equally in this work

*Corresponding author email: d.delneri@manchester.ac.uk

Fig. S1. ITS1 sequence alignment of *S. jurei* sp. nov., *S. cerevisiae*, *S. mikatae* and *S. paradoxus*. *S. jurei*-specific SNPs are highlighted in grey. The strain details are: Sc, *S. cerevisiae* NRRL Y-12632^{NT} (AY046146); Spd_Eur, *S. paradoxus* CBS 432^{T}, European population (AY046148); Spd_FE, *S. paradoxus* IFO 1804, Far Eastern population (AB533544); Spd_Am, *S. paradoxus* UFRJ 50816*, American population (AJ271809); Sj, *S. jurei* NCYC 3947^{T} (HG764814); Smk, *S. mikatae* NRRL Y-27341^{T} (AY046149). *Type strain of *S. cariocanus*.

```
Sc         AAGAAATTTAATAATTTTGAAAATGGATTTTTTTGTTTTGGCAAGAGCATGAGAGCTTTT    60
Spd_Eur    ...........................................G................    60
Spd_FE     ...........................................G................    60
Spd_Am     ............................................................    60
Sj         .....................................................--.....    58
Smk        .....................................................--.....    58

Sc         ACTGGGCAAGAAGACAAGAGATGGAGAGTCCAGCCGGGCCTGCGCTTAAGTGCGCGGTCT   120
Spd_Eur    .................................T..........................   120
Spd_FE     .................................T..........................   120
Spd_Am     .................................T..........................   120
Sj         ............T....................TG.........................   118
Smk        .................................TG.........................   118

Sc         TGCTAGGCTTGTAAGTTTCTTTCTTGCTATTCCAAACGGTGAGAGATTTCTGTGCTTTTG   180
Spd_Eur    ....T...................................................A...   180
Spd_FE     ....T...................................................A...   180
Spd_Am     ....T................................A..................A...   180
Sj         .A...................................A.........C.T....T.....   178
Smk        .....................................A................T.....   178

Sc         TTATAGGACAATTAAAACCGTTTCAATACAACACACTGTGGAGTTTTCATATCTTTGCAA   240
Spd_Eur    ............................................................   240
Spd_FE     C...........................................................   240
Spd_Am     ............................................................   240
Sj         ...............................................T............   238
Smk        ...........C...................................T............   238

Sc         CTTTTTCTTTGGGCATTCGAGCAATCGGGGCCCAGAGGTAACAAACACAAACAATTTTAT   300
Spd_Eur    ...........................A................................   300
Spd_FE     ...........................A................................   300
Spd_Am     ...........................A................................   300
Sj         ..............T............A................................   298
Smk        ..............T.............A...............................   298

Sc         CTATTCATTAAATTTTTGTCAAAAACAAGAATTTTCGTAACTGGAAATTTTAAAA-TATT   359
Spd_Eur    T......................................................A....   360
Spd_FE     T......................................................A....   360
Spd_Am     T......................................................A....   360
Sj         T......................................................A....   358
Smk        T......................................................A....   358

Sc         AA                                                             361
Spd_Eur    ..                                                             362
Spd_FE     ..                                                             362
Spd_Am     ..                                                             362
Sj         ..                                                             360
Smk        ..                                                             360
```

Table S1. Number of yeast isolates obtained from oak trees at different temperatures.

Sampling trees	30°C (Soil)	30°C (Bark)	20°C (Soil)	20°C (Bark)
Oak 1	15	None	20	None
Oak 2	10	None	25	None
Oak 3	13	35	20	74
Oak 4	14	None	26	None
Oak 5	11	None	21	None

Table S2. Nucleotide sequence similarity among *S. jurei* (Sj), *S. cerevisiae* (Sc), *S. mikatae* (Sm) and *S. paradoxus* (Sp).

Genes	Sj vs Sc	Sj vs Sm	Sj vs Sp	Sc vs Sp	Sc vs Sm	Sm vs Sp
CAT8	80%	92%	85%	87%	79%	84%
CYR1	82%	92%	85%	89%	81%	84%
OPY1	87%	92%	87%	92%	88%	88%
GSY1	87%	95%	89%	92%	87%	89%
MET6	90%	94%	90%	92%	90%	91%
TEF1	98%	98%	98%	99%	99%	99%
RPB2	88%	95%	89%	93%	89%	90%

Table S3. Physiological characteristics of *S. jurei* (Sj), *S. cerevisiae* (Sc), *S. mikatae* (Sm) and *S. paradoxus* (Sp).

**Fermentation**	**Sj**	**Sc***	**Sm***	**Sp***
Glucose	+	+	+	+
Galactose	+	v	+	+
Sucrose	+	v	+	+
Maltose	+	v	s	v
Raffinose	+	v	+	v
Melibiose	-	v	+	-
Melezitose	+	v	-	s
Methyl-α-D-glucoside	+	v	+	v
**Assimilation**				
Glucose	+	+	+	+
Sucrose	+	v	+	+
Raffinose	+	v	+	+
Melibiose	-	v	+	-
Galactose	+	v	+	+
Trehalose	d	v	s	v
Maltose	+	v	+	v
Melezitose	+	v	+	v
Methyl-α-D-glucoside	+	v	+	v
Ethanol	+	v	+	+
Glycerol	d	v	-	v
Ribitol	-	-	+	-
Galactitol	-	-	+	-
D-Mannitol	+	-	+	+
Lactate	+	v	+	+
**Additional Test**				
Growth at 37˚C	-	v	-	v

Growth test results: +, positive; s, slow; d, delayed; -, negative; v, variable

*** Data taken from**: (1, 2)

References

1. Barnett JA, Payne RW, Yarrow D. Yeasts: Characteristics and Identification, 3rd edn. Cambridge Academic Press. 2000.

2. Vaughan-Martini A, Martini A. Saccharomyces Meyen ex Reess (1870). In The Yeasts, A Taxonomic Study, 5th edn. Pp 733-746. Edited by C.P. Kurtzman, J.W. Fell & T. Boekhout. Amsterdam: Elsevier. 2011.
